# Olfactory dysfunction as a prognostic marker for disability progression in Multiple Sclerosis: An olfactory event related potential study

**DOI:** 10.1371/journal.pone.0196006

**Published:** 2018-04-17

**Authors:** Rosella Ciurleo, Lilla Bonanno, Simona De Salvo, Laura Romeo, Carmela Rifici, Edoardo Sessa, Giangaetano D’Aleo, Margherita Russo, Placido Bramanti, Silvia Marino, Fabrizia Caminiti

**Affiliations:** 1 IRCCS Centro Neurolesi “Bonino-Pulejo”, Messina, Italy; 2 Department of Biomedical and Dental Sciences and Morphological and Functional Imaging, University of Messina, Messina, Italy; National Institutes of Health, UNITED STATES

## Abstract

Multiple sclerosis (MS) is a chronic inflammatory disease and one of the leading causes of disability in young adults. Functional markers able to predict MS progression are still lacking. It is recognized that olfactory dysfunction may be an early symptom in MS. The aim of this study was to investigate whether alterations in olfactory event-related potentials could play a prognostic role in MS. Thirty patients affected by MS relapsing-remitting underwent an olfactory potential examination (T0). Three years after baseline (T1), 28 of 30 patients were clinically evaluated by expanded disability status scale. In addition, the number of Disease Modifying Therapies (DMTs) and the total number of relapses occurred in the last 3 years were collected. At T1, we observed a negative correlation between presence/absence of olfactory potentials and expanded disability status scale scores (rpb = -0.48; p = 0.009). A significant trend for a negative correlation between presence/absence of olfactory potentials and disease duration (rpb = -0.36; p = 0.06) and total number of relapses (rpb = -0.34; p = 0.08) was found. Only patients with olfactory potential absence showed a significant trend in the difference of the disability status scale (p = 0.06) between T0 and T1. In the sub-group of patients with reduced olfactory potential amplitude, we detected a trend for a negative correlation between the disability status scale and the amplitude of N1-P2 components more marked at T1 (r = -0.52; p = 0.06) than T0 (r = -0.47; p = 0.09). This is the first study that evaluated the prognostic role of olfactory event-related potentials in MS. Our results highlighted that olfactory alterations of MS patients were related to disability progression and, to a lesser extent, disease activity. The analysis of olfactory potential parameters confirmed the involvement in olfactory network damage of inflammatory and/or neurodegeneration processes which could predict the progressive course of the disease.

## Introduction

Multiple Sclerosis (MS) is a chronic autoimmune progressive disease of central nervous system where olfactory dysfunctions have recently been described [1–4], with a prevalence from 20% to 45% [5].

It has been reported that olfactory dysfunction may be considered a marker of disability progression in MS. Indeed, several studies [6–8] found a correlation between olfactory dysfunctions and higher Expanded Disability Status Scale (EDSS) scores and longer disease duration (DD) in MS patients, highlighting the potential role of smell assessment in monitoring of MS evolution. However, these studies have used psychophysical tests of olfactory function. Among these, one of the best-validated olfactory tests is the Connecticut Chemosensory Clinical Research Center Test (CCCRC Test). This consists of smell threshold test and smell identification test. The threshold test uses 11 different concentrations of n-butanol in separate glass flasks. The patient is asked to identify the strongest smell among the flask containing the lowest concentration of n-butanol and a flask with water. If the answer is wrong, the patient is offered the next highest concentration flask, and so on until exact response. In identification test, 7 flasks containing different substances are presented to the patient. The patient is asked to select the stimulus name from a list of 7 odors [9]. The psychophysical olfactory tests do not ensure an objective smell assessment, especially if the cognitive functions and attention levels are impaired, as it occurs both in early and in advanced MS. In addition, these olfaction studies in MS have not a longitudinal design able to evaluate the correlation over time between olfactory dysfunction and disability progression.

Olfactory Event-Related Potentials (OERPs) are a valid electrophysiological technique to assess olfactory system. This method allows an objective evaluation of changes in olfactory function. Indeed, it is independent from patients’ response bias. OERP presence is a strong indicator of good olfactory function; conversely, the OERP absence suggests an olfactory loss. OERPs are the result of sequential activation of different brain areas that begins from olfactory bulbs and tracts and involves the orbitofrontal and insular cortices, along with rostrum-medial regions of the temporal lobe [10].

To date, there is an unmet need for markers predicting long-term disability in MS patients. To our knowledge, no study has explored the prognostic value of OERPs in predicting long-term disability in MS.

The aim of this longitudinal study was to investigate the relationship between the OERP alterations, disease activity and disability progression in Relapsing Remitting MS (RRMS) patients, whose total number of relapses (TNR) and EDSS were measured three years after the evaluation of olfactory function.

## Materials and methods

Thirty patients (19 females and 11 males) with diagnosis of RRMS according to the revised McDonald criteria [11] (mean age of 36.03±66.96 years and mean EDSS score of 2.08±61.07) were recruited from June to October 2013 at IRCCS Centro Neurolesi “Bonino-Pulejo” of Messina, Italy. The DD ranged from 2 to 13 years with a mean duration of 5.87±3.29 years [3].

At baseline (T0), all MS patients underwent an OERP examination to evaluate their olfactory function [3]. As previously reported, 7 of 30 MS patients showed a marked olfactory dysfunction, as detected by OERP absence, while 23 patients showed OERP responses. Of these latter, 16 had a strong reduction of N1-P2 component amplitude, but normal latency (borderline OERPs), compatible with a mild olfactory impairment, and the remaining 7 patients had normal latency and amplitude of N1 and P2 components (normal OERPs), compared with healthy controls [3].

Three years after baseline (T1), 28 of 30 patients were clinically evaluated. Two MS patients drop-out follow-up. Based on the previous OERP results, we divided the remaining 28 MS patients into two groups: group with OERP absence and group with OERP presence. This group was divided into two further sub-groups: the group with normal OERPs and the group with borderline OERPs. At visit T1, patients’ clinical characteristics, such as EDSS score, number of Disease Modifying Therapies (DMTs) changed and TNR occurred in the last 3 years, were collected. The EDSS evaluations were conducted by Neurostatus certified rater.

The study was conducted in accordance with the Declaration of Helsinki and was approved by Ethic Committee of IRCCS Centro Neurolesi “Bonino-Pulejo”; all patients gave written informed consent before any study-related procedures were performed.

## Statistical analysis

The analysis was conducted with a descriptive statistic of sociodemographic and clinical characteristics of groups. The statistical relationship between presence/absence of OERPs (dichotomous variable), and EDSS scores, DD and TNR was evaluated by the point-biserial correlation coefficient. Normal distribution of data was evaluated by using the Shapiro-Wilk normality test. The Wilcoxon signed-rank test was used to compare the relationship among the EDSS scores collected at T0 and T1 in each group (intra-group analysis). For inter-group analysis, Mann-Whitney-U test was used to highlight the differences between the groups at T0 and T1. Spearman’s rank correlation coefficient was used in OERP absence and presence group, in order to assess whether there was a relationship among number of DMTs, EDSS scores and TNR. Correlations between the EDSS scores and OERP parameters were computed by Spearman’s coefficient in the group with normal and borderline OERPs (intra-group analysis). Statistical analyses were performed using an open source R3.0 software package. A 95% of confidence level was set with a 5% alpha error. Statistical significance was set at p<0.05.

## Results

### Sample analysis

The demographic and clinical characteristics and the main parameters of OERP components of MS patients are showed in Table 1. At T1, we observed a negative correlation between presence/absence of OERPs and EDSS scores (rpb = -0.48; p = 0.009): higher EDSS scores was related to OERP absence. In addition, we found a significant trend for a negative correlation between presence/absence of OERPs and DD (rpb = -0.36; p = 0.06) and TNR (rpb = -0.34;p = 0.08).

**Table 1:** Socio-demographic and clinical characteristics and main parameters of OERP components of groups at T1, frequencies (%).

					OERP Presence	
	All	OERP Presence (T0)	OERP Presence	OERP Absence	Borderline OERPs	Normal OERPs
Number of Patients	28 (100%)		21 (75%)	7 (25%)	14 (67%)	7 (33%)
Age (mean±SD)	43.07 ± 10.09		39.71 ± 8.14	53.14 ± 8.93	41.43 ± 8.60	36.28 ± 6.32
EDSS (T1) (mean±SD)	2.62 ± 1.68		2.17 ± 1.33	4.0 ± 1.96	2.03 ± 1.36	2.43 ± 1.34
DD (mean±SD)	5.89 ± 3.40		5.19 ± 3.64	8.0 ± 1.0	5.5 ± 3.84	4.57 ± 3.41
TNR (mean±SD)	1.32 ± 1.19		1.09 ± 1.04	2.0 ± 1.41	0.93 ± 1.07	1.43 ± 0.97
Number of DMTs	1.30 ± 1.30		1.19 ± 0.98	1.64 ± 2.05	1.07 ± 1.07	1.43 ± 0.79
N1-Latency (mean±SD)	637.22 ± 27.49	637.39 ± 23.77	637.22 ± 27.49	-	640.22 ± 29.64	631.38 ± 23.16
P2-Latency (mean±SD)	723.55 ± 21.68	637.39 ± 23.77	723.55 ± 21.68	-	728.63 ± 22.25	713.62 ± 16.93
P2-N1 Amplitude (mean±SD)	3.75 ± 1.87	637.39 ± 23.77	3.75 ± 1.87	-	2.90 ± 0.95	5.41 ± 2.11

Legend: DD years = Disease Duration; DMTs = Disease Modifying Therapies; EDSS = Expanded Disability Status Scale; SD = Standard Deviation; TNR = Total Number of Relapses

Latency values are in ms. Amplitude values are in mV.

#### Intra- and inter-group analysis

**Fig 1:**
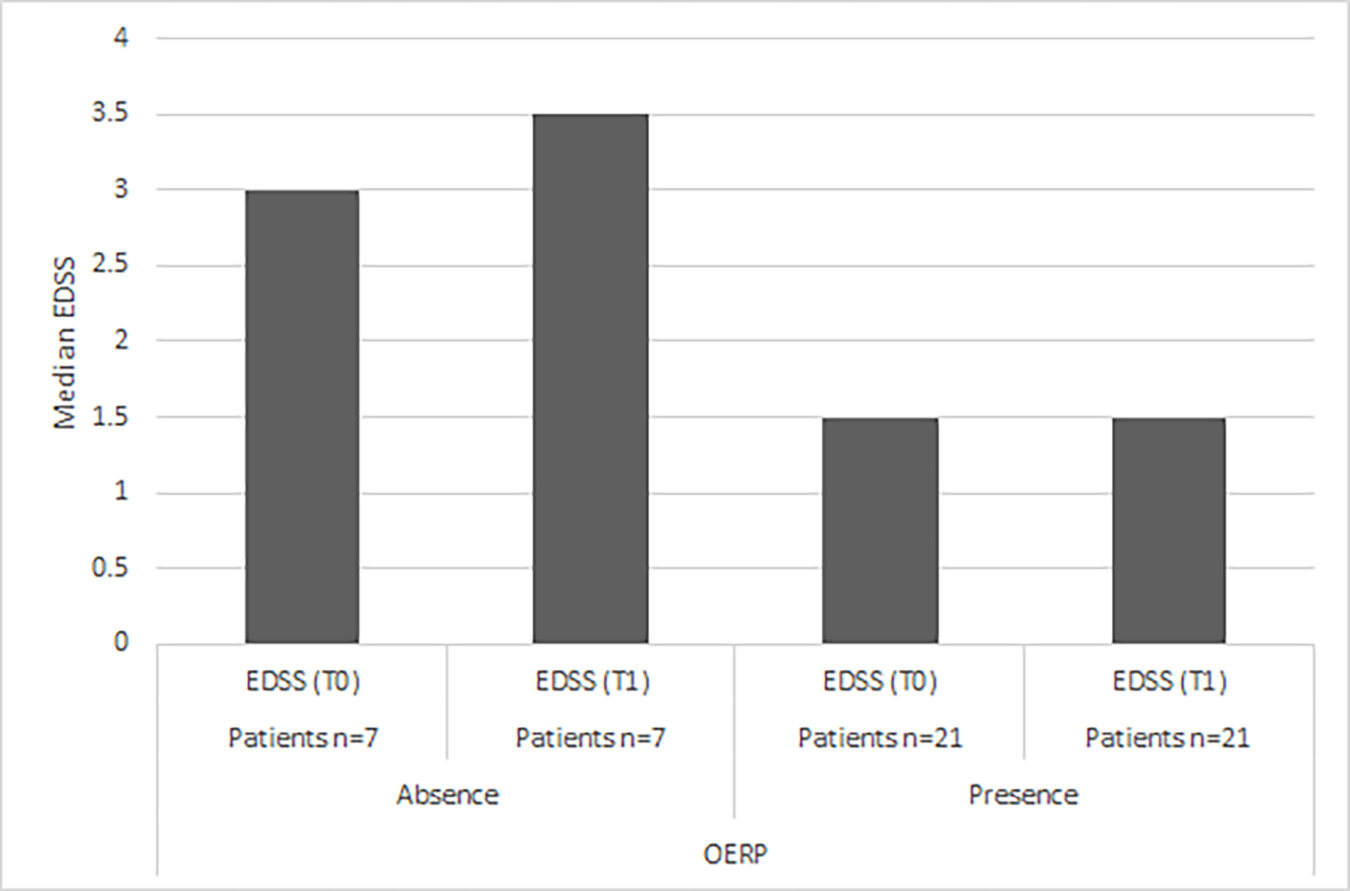
EDSS scores in patients with OERP absence and presence at T0 and T1.

In the group with OERP absence, the difference in EDSS scores (p = 0.06) between T0 and T1 showed a significant trend. In the group with OERP presence, no statistical difference in EDSS scores (p = 0.49) among two times was found (Table 2) (Fig 1). No significant correlation between number of DMTs with EDSS (p = -0.54; p = 0.21 in group with OERP absence; p = -0.48; p = 0.27 in group with OERP presence) and TNR (p = -0.16; p = 0.73 in group with OERP absence; p = -0.03; p = 0.95 in group with OERP presence) was observed. In the same way, no statistical difference were showed in EDSS scores in group with borderline OERPs (p = 0.72) and in group with normal OERPs (p = 0.09) between T0 and T1 (Table 3).

**Table 2:** Intra and inter-group analysis in the groups with absence and presence of OERPs.

		Absent Median (first-third quartile)	Present Median (first-third quartile)	p-value *(U-Mann Whitney)*
DD	T0	5 (4.5–5.0)	0 (-1.0–6.0)	0.11
	T1	8 (7.5–8.0)	3 (2.0–9.0)	0.11
	p-value *(Wilcoxon)*	0.01*	0.0001**	-
EDSS	T0	3 (2.2–3.0)	1.5 (1.0–2.0)	0.13
	T1	3.5 (3.0–4.7)	1.5 (1.0–2.5)	0.02*
	p-value *(Wilcoxon)*	0.06	0.49	
TNR	T0	NA	NA	**-**
	T1	2.0 (1.0–2.0)	1.0 (0–2.0)	0.14
	p-value *(Wilcoxon)*	-	-	-

Legend: DD years = Disease Duration; EDSS = Expanded Disability Status Scale; TNR = Total Number of Relapses

*p <0.05

**p<0.001

**Table 3:** Intra and inter-group analysis in the groups with borderline and normal OERPs.

		Borderline OERPs Median (first-third quartile)	Normal OERPs Median (first-third quartile)	p-value *(U-Mann Whitney)*
DD	T0	1 (0–6.5)	0 (0–3.0)	0.37
	T1	4.0 (2.2–9.5)	3.0 (2.5–6.0)	0.54
	p-value *(Wilcoxon)*	<0.001*	<0.001*	-
EDSS	T0	1.5 (1.0–2.0)	1.5 (1.25–2.7)	0.53
	T1	1.2 (1–2.4)	2.0 (1.5–3.2)	0.38
	p-value *(Wilcoxon)*	0.72	0.09	-
TNR	T0	NA	NA	-
	T1	0.5 (0–2.0)	1.0 (1.0–2.0)	0.3
	p-value *(Wilcoxon)*	-	-	-

Legend: DD years = Disease Duration; EDSS = Expanded Disability Status Scale; TNR = Total Number of Relapses

*p<0.001

In the inter-group analysis, at T1 we observed a significant difference between two groups with and without OERP_S_ in EDSS scores (p = 0.02), but no in DD (p = 0.11) and TNR (p = 0.14) (Table 2). No significant difference in all variables between the groups with normal and borderline OERPs was found at T1 (EDSS: p = 0.15; DD: p = 0.38; TNR: p = 0.54) (Table 3).

### Spearman correlation at T0 and T1

In the group with borderline OERPs, we detected a trend for a negative correlation between the EDSS scores and the amplitude of N1-P2 components in Cz (r = -0.47; p = 0.09) at T0 and a more marked trend for a negative correlation among the same variables (r = -0.52; p = 0.06) at T1 (Table 4). In the group with normal OERPs, no significant correlation between EDSS scores and OERP parameters at T0 and T1 was found (Table 4).

**Table 4:** Correlation between EDSS scores and OERP parameters in groups with borderline and normal OERPs.

	Borderline OERPs				Normal OERPs			
	EDSS (T0)		EDSS (T1)		EDSS (T0)		EDSS (T1)	
	R	p	r	p	r	p	r	p
N1-Fz latency	-0.11	0.69	-0.1	0.73	0.4	0.37	0.31	0.5
N1-Cz latency	0.06	0.85	0.05	0.85	0.21	0.65	0.25	0.58
N1-Pz latency	0.08	0.79	0.02	0.95	0.009	0.98	0.02	0.97
P2-Fz latency	-0.15	0.61	-0.21	0.46	0.56	0.19	0.56	0.19
P2-Cz latency	0.13	0.67	0.15	0.61	0.61	0.14	0.49	0.26
P2-Pz latency	0.13	0.67	0.08	0.78	0.5	0.25	0.4	0.37
P2-Fz amplitude	-0.009	0.97	-0.1	0.73	-0.1	0.83	-0.07	0.88
P2-Cz amplitude	-0.47	0.09	-0.52	**0.06**	0.3	0.51	0.28	0.54
P2-Pz amplitude	-0.09	0.76	-0.18	0.56	0.14	0.77	0.3	0.51

Legend: EDSS = Expanded Disability Status Scale

## Discussion

MS is a chronic disease with a variable and unpredictable course that may cause severe disability over time. Despite the recent advances in the understanding of the disease and in the introduction of therapeutic interventions, prognostic markers are still lacking. The identification of markers able to predict a favorable or poor outcome would be useful in order to detect patients at a higher risk of disease progression and, then, to plan a more suitable therapeutic management for the patient.

The aim of this longitudinal study was to investigate the role of OERPs as marker predicting clinical outcomes in MS. Indeed, we evaluated the disability progression of MS patients, measured as change of EDSS scores from T0 to T1 (3 years after), and its relationship with olfaction performances, detected by using OERPs at T0.

In our previous study [3], we observed a clear association of the disability degree and DD with the olfactory dysfunction. Indeed, MS patients with higher EDSS scores and DD showed marked smell alterations, observed by OERP absence.

In the present follow-up study, we found that MS patients who had not shown OERPs at baseline, 3 years after the OERP examination (T1), showed an increase in EDSS scores, while the patients who had shown OERPs had the EDSS scores unchanged. The EDSS scores collected at T1 were higher in the MS patients without OERPs compared to patients with OERPs. On the contrary, the DD and TNR were similar among the patients with and without OERPs. The increase of EDSS scores in MS patients with OERP absence as well as the unchanging EDSS scores in MS patients with OERP presence did not seem depend from number of DMTs changed in last 3 years.

In addition, by correlation analysis we found that OERP absence were related to probability to detect higher EDSS scores and, in a more moderate way, higher DD and TNR at T1. We observed that in patients with borderline OERPs there was a relationship between reduced amplitude of N1-P2 components and higher EDSS scores collected at T1.

The patients without OERPs were, on average, 12 years older than the patients with OERPs and had a longer DD. In addition, patients with reduced amplitude were older than patients with normal amplitude. Our MS patient sample consisted of young adults (average age: 43.07±10.09 years). Within the age range of the patients in our study, it is difficult to distinguish disease related changes in olfactory performance from those associated with advanced age. Indeed, olfactory decline is present in over half of subjects between the ages of 65 and 80 years and in over three quarters of those over the age of 80 years. Therefore, the dysfunction of smell found in a MS patient group is probably related to disease progression, as confirmed by EDSS scores. Moreover, a longer DD in MS patients without OERPs confirms that the olfactory dysfunction correlates positively with disability level.

Other non-longitudinal studies reported a relationship between olfactory dysfunction and disability progression. In particular, a cross-sectional study [7] found olfactory dysfunction in 32% of 100 MS patients evaluated by using the Connecticut test. In particular, patients with EDSS above 4.0 showed an increased risk of olfactory dysfunction. Another study [6] performed in 153 MS patients showed that 11.1% of them had olfactory dysfunction. This dysfunction was higher in secondary progressive MS than in RRMS with good correlation between olfactory impairment and longer DD. Li-Min and colleagues [8] demonstrated that olfactory detection and recognition threshold were significantly higher in MS patients than healthy controls. In addition, recognition threshold correlated positively with EDSS.

To our knowledge, in the literature there is a single longitudinal study evaluating the olfactory function in MS patients [12]. In sample of MS patients, including relapsing-remitting and primary progressive forms, no change in olfactory function, assessed by using psychophysical test, and in physical disability, expressed in EDSS score, was found over time. It has been reported only a correlation between the TNR and a reduced capacity of odor discrimination.

In our longitudinal study, we found a worsening of the disability over time which was more marked in MS patients who have previously shown altered olfactory potentials, supporting the prognostic role of OERPs in MS. In addition, also the moderate association between poor olfactory function and disease activity, as demonstrated by TNR, confirmed that olfaction disturbances could anticipate the future disability.

OERP latency and amplitude characteristics may provide an indication of pathological conditions of olfactory networks in MS. Latency prolongation is due to demyelination, while reduced amplitude is related to conduction block or axonal loss [13]. Indeed, the amplitude reduction is function of the reduction of stimulated cell number. Then, we can argue that the neural degeneration in MS compromises the connecting resources which are not able to induce a cortical recruitment of fibers useful for a complete generation of the amplitude wave.

It is now well known that MS is not only an inflammatory disease but also a neurodegenerative disease involving axonal transection and neuronal damage [14]. In MS the axonal neurodegeneration is considered a direct cause of progressive disability [15]. Currently, increasing evidence indicates that the axonal degeneration in MS already begins at disease onset [16] and may occur independently from inflammatory destruction of myelin [14,17,18].

Our OERP results lead us to speculate that central olfactory networks of MS patients with complete impairment of olfaction function (OERP absence) could be damaged by both neurodegeneration and inflammatory activity. Indeed, in these patients we found not only a good relationship between marked smell alterations and disability progression, detected by EDSS, but also a trend correlating the smell alterations with the signs of disease activity, detected by TNR. The mutual influence of these processes continuing over time, neurodegeneration and inflammation, could be responsible for marked olfaction dysfunctions which, evaluated in good time, could are able to predict the poor clinical outcome in MS. On the contrary, it is likely that in damage of central olfactory structures of MS patients with borderline OERPs, in which only the amplitude is altered, is involved a neurodegeneration process that, occurring earlier and independently from the inflammatory process, could be responsible for a more favorable clinical outcome. However, this hypothesis requires both a Magnetic Resonance Imaging study and a longer follow-up study assessing over time not only disability progression but also olfactory function by using OERPs as well as their relationship. However, the association between disability degree and OERP amplitude reduction, found in our study, supports the importance of the analysis of OERP component parameters in order to achieve an objective data predicting MS progression.

In conclusion, this is the first longitudinal study which evaluated the prognostic role of olfactory function by using OERPs in MS patients. The encouraging results are a first potential indication of how olfactory performances, very often underestimated by the clinicians, may predict over time the MS evolution. In particular, OERPs might be: i) for their reliability, validity and quantifiability, good candidates as biomarkers to select patient groups at high risk of disease progression for phase II clinical trials; ii) for their tolerability and efficiency, an useful additional tool in clinical protocols not only to early diagnose MS but also to predict its course.
